# *Desmodesmus* Extract as a Mitochondrion-Targeted Neuroprotective Agent in Parkinson’s Disease: An In Vitro Study

**DOI:** 10.3390/cimb47030174

**Published:** 2025-03-06

**Authors:** Muazzez Derya-Andeden, Pinar Altin-Celik, Enver Ersoy Andeden, Hamiyet Donmez-Altuntas

**Affiliations:** 1 Department of Medical Biology, Faculty of Medicine, Erciyes University, 38030 Kayseri, Türkiye; pnar.altnclk@gmail.com (P.A.-C.); donmezh@erciyes.edu.tr (H.D.-A.); 2 Department of Molecular Biology and Genetics, Faculty of Art and Science, Hacı Bektaş Veli University, 50300 Nevşehir, Türkiye; enverandeden@nevsehir.edu.tr

**Keywords:** Parkinson’s disease, microalgae, SH-SY5Y, 6-OHDA, mitochondrial damage, oxidative stress

## Abstract

Parkinson’s disease (PD) is the second most common neurodegenerative disease, and its prevalence is expected to double in the next 30 years. Currently, no effective treatment exists for Parkinson’s disease. Thus, the research has focused on discovering new natural compounds with strong neuroprotective potential. This study aimed to investigate the effects of the methanol extract of *Desmodesmus arthrodesmiformis* EM13 (DaMe) on the mitochondrial damage pathway in an in vitro model of PD. The isolate of *Desmodesmus arthrodesmiformis* EM13 was first grown under appropriate culture conditions, and then the extract (DaMe) was prepared for use in the experiments. The total lipid and protein contents, fatty acid composition, and elemental content of DaMe were subsequently determined. Human SH-SY5Y neuroblastoma cells were pretreated with nontoxic concentrations of DaMe before 6-hydroxydopamine (6-OHDA) toxicity. Pretreatment with DaMe at concentrations of 100, 250, and 500 µg/mL showed a neuroprotective effect on 6-OHDA-induced SH-SY5Y neuroblastoma cells by decreasing the reactive oxygen species (ROS) production, decreasing the total oxidant status (TOS), increasing the total antioxidant capacity (TAC), increasing the mitochondrial membrane potential (ΔΨm), decreasing the oxidative DNA damage, and regulating gene expressions related to PD and apoptosis. Given the results of our study, we suggest that DaMe can be used as a natural source for producing drugs and dietary supplements intended to treat PD.

## 1. Introduction

Parkinson’s disease (PD) is the second most common neurodegenerative disease, affecting approximately ten million people worldwide. It typically occurs around the age of sixty, with complex interactions between aging, genetics, and the environment playing a role [1]. One of the main causes of neurodegeneration in PD is oxidative stress. While oxidative stress is caused by the increased production of reactive oxygen species (ROS), the physiological levels of these molecules are a normal byproduct of metabolic processes and are critical for cellular defense [2]. During oxidative stress, DNA’s hydroxyl radicals and guanine residues oxidize and form 8-hydroxy 2-deoxyguanosine (8-OHdG), a marker of DNA damage [3]. Compared with those in normal controls, the 8-OHdG concentrations in both the substantia nigra (SN) and the cerebrospinal fluid (CSF) are significantly elevated in patients with PD [4,5]. One of the most important pathological features of PD has long been assumed to be mitochondrial dysfunction. Since mitochondria are extremely adaptable organelles, the survival and function of neurons depend on their integrity [6].

Current pharmacological treatments for PD fail to halt or decelerate its progressive neurodegeneration, providing only modest and transient symptomatic relief, whereas prolonged use of these treatments is associated with significant adverse effects [7]. To date, no new pharmacologically effective treatment has been developed to prevent or alleviate the progression of PD. Owing to the sharp increase in the mortality and morbidity rates in this disease, there is a need to develop more effective treatments. Therefore, to understand the pathophysiological mechanisms of PD and develop disease-modifying treatments, the research is continuing to discover new therapeutic molecules, preferably of natural origin, with strong neuroprotective potential and few or no side effects [8]. Numerous studies have attempted to identify medications to inhibit the progression of PD, and new compounds are under development [9].

The use of molecules with different properties and activities extracted from various living species via traditional medicine as active pharmaceutical ingredients plays an important role in medicine and pharmacy. In this context, microalgae are powerful biomolecule sources that have various pharmacological activities and attract great attention in the fields of biotechnology, energy, food, medicine, and cosmetics owing to the presence and diversity of secondary metabolites such as proteins, pigments, and carotenoids, polyphenols, sterols, polyunsaturated fatty acids, and polysaccharides that they secrete at different stages of growth [10]. Since they contain different molecules and have various biological activities to adapt to environmental conditions, it is believed that the extracts obtained from some microalgae and/or the biomolecules isolated from the extracts could have a therapeutic profile in the treatment of PD and provide alternative contributions to various disciplines of health sciences.

*Desmodesmus* is a genus of green microalgae commonly found in aquatic environments [11]. Owing to its high productivity, ease of cultivation, and potential as a source of high-quality fatty acids, it is of interest as a material for biofuels [12,13]. In our previous study, we demonstrated that the methanol extract of *Desmodesmus arthrodesmiformis* EM13 (DaMe), which was prepared from *Desmodesmus arthrodesmiformis* EM13 isolated from a natural source in Türkiye (Kızılırmak River, Nevşehir) and characterized both morphologically and molecularly, has a high antioxidant capacity and neuroprotective effect against 6-hydroxydopamine (6-OHDA) toxicity in human SH-SY5Y neuroblastoma cells due to its antiapoptotic properties. In the same study, seven phenolic molecules (caffeic acid, gallic acid, quercetin dihydrate, vanillic acid, hesperidin, salicin, trans-resveratrol) and sixteen medicinally active substances (alprazolam, d-amphetamine, d-methamphetamine, caffeine, 3,4-methylenedioxy-methamphetamine (d. I-MDMA), diclofenac, atenolol, warfarin, diazepam, clonazepam, oxazepam, sulfamethoxazole, temazepam, naproxen, ibuprofen, acetylsalicylic acid) in DaMe were quantified by LC/ESI-MS/MS analysis [14]. With the results of our previous study mentioned, the invention titled ‘Use of methanol extract of *Desmodesmus arthrodesmiformis* EM13 as an active substance reservoir in drug research and development’ has been registered as a PATENT with an examination by the TURKISH PATENT AND TRADEMARK OFFICE (patent number: TR 2022 017725 B).

In this study, we investigated the impact of this extract on the mitochondria and oxidative DNA damage in the experimental PD model. This is the first study to examine the therapeutic effect of *Desmodesmus* sp. extract on the mitochondrial damage pathway in an in vitro model of PD.

## 2. Materials and Methods

### 2.1. Microalga Cultures

A pure isolate of the green freshwater microalga *Desmodesmus arthrodesmiformis* EM13 (GenBank accession number: OM728838) was kindly provided by Prof. Dr. Şahlan ÖZTÜRK (Nevşehir Hacı Bektaş Veli University, Türkiye). A total of 20 mL of the stock culture containing late-logarithmic-phase cells was inoculated into 200 mL of sterile BG-11 medium, and the cultures were incubated in an incubator under the following conditions: 23 °C, a 12:12 h light/dark photoperiod, and shaking at 90 rpm.

### 2.2. Preparation of the Extract (DaMe)

The microalga cultures were harvested by centrifugation at room temperature (4000 rpm, 10 min) together with the culture medium. The resulting pellets were then freeze-dried for 48 h at −85 °C under a pressure reduction from 5.00 mbar to 0.01 mbar. For extraction, 10 mg of freeze-dried microalgae was weighed, ground with a glass rod, and mixed with 2 mL of methanol. The mixture was incubated on a shaker at room temperature (250 rpm) for 24 h to facilitate extraction. After extraction, the mixture was centrifuged at 4000 rpm for 10 min. The supernatant was collected in a new tube and the remaining pellet was re-extracted by adding methanol; this process was repeated two more times. The supernatants from all extraction steps were combined and passed through a 0.22 μm filter to remove all particulates. The filtered extract was then concentrated via a rotary evaporator at 45 °C for 3.5 h under reduced pressure. The resulting dry extract (DaMe) was dissolved in dimethyl sulfoxide (DMSO) to a final concentration of 2 mg/mL and stored in aluminum foil at −20 °C in the dark until further use.

### 2.3. Quantification of the Total Lipid and Protein Contents of DaMe

The total lipid content was detected using the sulfo-phospho-vanillin (SPV) method [15]. One milliliter of cell suspension was centrifuged, and the supernatant was subsequently discarded. The remaining cell pellet was suspended in 100 μL of pure water. The total lipid quantification was subsequently performed via the SPV method. The absorbance readings were taken at 530 nm. The standard curve was prepared from OD data from five different concentrations of canola oil. To estimate the protein content of the dried biomass, protein extraction was performed via incubation in 24% (*w*/*v*) TCA at 95 °C for 15 min followed by hot alkaline treatment for 2 h [16]. The protein content in the protein extract was quantified following the modified Lowry method [17]. The standard curve was generated from OD data from five different concentrations of bovine serum albumin (BSA). The results were expressed as percentages of dry extract (μg/mL).

### 2.4. The Fatty Acid Composition and the Elemental Content of DaMe

The fatty acid composition of DaMe was determined via gas chromatography–mass spectrometry (GC-MS) and the elemental content was determined via the inductively coupled plasma–mass spectrometry (ICP-MS) method at the Erciyes University Technology Research and Application Center as a service.

### 2.5. Cell Culture and In Vitro Model of PD

The human neuronal SH-SY5Y cell line was purchased from the American Type Culture Collection (ATCC) and grown in Dulbecco’s modified Eagle medium (DMEM) (Gibco, Thermo Fisher Scientific, Winsford, UK) containing L-glutamine (2 mM), high glucose (4500 µg/mL), and NaHCO_3_ (22 mM) supplemented with 10% fetal bovine serum (FBS) (Biological Industries, Kibbutz Beit-Haemek, Israel), penicillin (100 U/mL), streptomycin (100 µg/mL), and 40% endothelial basal medium MCDB-107 (Life Science, Berlin, Germany) at 37 °C under a humidified atmosphere of 95% air and 5% CO_2_. The SH-SY5Y cells were used below passage 12 for all the experiments to avoid phenotypic changes or cellular senescence. As determined in our previous study, 150 µM, which is the 50% inhibitory concentration (IC_50_) value for 6-OHDA, and 100, 250, and 500 μg/mL for nontoxic concentrations of DaMe were used for the SH-SY5Y cell treatment for 24 h [14].

### 2.6. Intracellular ROS Production

The level of intracellular ROS generation was determined via the use of 2′,7′-dichlorofluorescein diacetate (DCFH2-DA), a cell-permeable fluorescent probe. The cells were seeded at a density of 1 × 10^6^ cells per well in a 6-well plate. The samples were pretreated with DaMe (100, 250, and 500 μg/mL) for 24 h. Then, toxicity was induced with 150 μM of 6-OHDA for an additional 24 h. The control group included non-treated cells. After incubation, the medium was removed, and the cells were collected and treated with 10 μM DCFH-DA for 30 min at 37 °C. The fluorescence intensity was measured using a microplate reader at 485 nm for excitation and 530 nm for emissions. The results are expressed as the relative fluorescence unit (RFU) of each group. The 6-OHDA group was compared with the control group, and the DaMe groups were compared with the 6-OHDA group.

### 2.7. Total Oxidant Status (TOS) and Total Antioxidant Status (TAS)

The total oxidant status (TOS) and total antioxidant status (TAS) of the cells were measured spectrophotometrically according to the manufacturer’s instructions using the commercial kits (No: E-BC-K802-M, Elabscience, Houston, TX, USA and No: K025, Finetest, Wuhan, China, respectively). The cells were seeded at a density of 5 × 10^5^ cells per well in a 6-well plate and pretreated with DaMe (100, 250, and 500 μg/mL) for 24 h. Then, toxicity was induced with 150 μM 6-OHDA for an additional 24 h. The control group included non-treated cells. After trypsinization, the pellet was dissolved in water and crushed. It was kept on ice for 10 min and then centrifuged. Supernatants were transferred to a new 96-well plate for absorbance readings. The 3-ethylbenzothiazoline-6-sulfonate (ABTS) compound was colored; the TAS measurement measured how well the antioxidants in the SH-SY5Y cells converted it to a colorless form. At 660 nm, the reduction process was observed, and the results were expressed in mmol Trolox Equiv./L. Spectrophotometric measurement of the color density of the molecule formed in the reaction medium during the oxidation of ferrous ions to ferric ions at 530 nm quantifies the TOS present in the cells. The results were expressed as μmol H_2_O_2_ equiv./L. The oxidative stress index (OSI) was calculated by following Formula (1) [18]:

(1) $$OSI\;\left(arbitary\;unit\right)\left(\frac{TOS\;\left(\mu mol\;H_{2}O_{2}\frac{Eq}{L}\right)}{TAC\;\left(\mu mol\;Trolox\frac{Eq}{L}\right)}\right)\times 100$$

### 2.8. Mitochondrial Membrane Potential (ΔΨm)

The mitochondrial membrane potential was determined using a commercial kit (No: MAK159, Sigma-Aldrich, Saint Louis, MO, USA). The cells were seeded at a density of 5 × 10^4^ cells per well in a black 96-well plate. The samples were pretreated with DaMe (100, 250, and 500 μg/mL) for 24 h. Then, toxicity was induced with 150 μM of 6-OHDA for an additional 24 h. The control group included non-treated cells. According to the manufacturer’s recommended protocol, JC-10 dye was added to each well. After incubation for 45 min in the dark at 37 °C, assay buffer was added, and two separate fluorescence readings were taken using a microplate reader at 490 and 540 nm for excitation and 525 and 590 nm for emissions. The red (from first reading)/green (from second reading) ratio was used to determine the ΔΨm.

### 2.9. Oxidative DNA Damage

An 8-OHdG ELISA kit (Fine Test, #EU2548, Wuhan Fine Biotech Co., Ltd., Wuhan, China) was used for the quantitative detection of the 8-OHdG levels in the supernatant of the cells. The cells were seeded at a density of 5 × 10^5^ cells per well in a 6-well plate. After the cells were treated with DaMe (100, 250, and 500 μg/mL) for 24 h, toxicity was induced with 150 μM of 6-OHDA for an additional 24 h. After incubation, the medium was collected and centrifuged at 1500 rpm at 4 °C for 10 min. The supernatant was used to perform the ELISA according to the manufacturer’s protocol. The target concentration of the samples was interpolated from the standard curve obtained from the measurement of absorbance at 450 nm.

### 2.10. RT-qPCR Analysis

SH-SY5Y cells were seeded in a 6-well plate at a density of 1 × 10^7^ cells per well and pretreated with three concentrations of DaMe (100, 250, and 500 μg/mL) 24 h before exposure to 6-OHDA (150 μM) to evaluate the effects of the extract pretreatment on the expression of PD and apoptosis-related genes. The Parkinson’s-related synuclein alpha (*SNCA*), leucine-rich repeat kinase 2 (*LRRK2*), PTEN-induced kinase 1 (*PINK1*), Parkinson-associated protein deglycase 1 (*DJ-1*), and Parkin (*PARK2*) genes and the apoptosis-related *p53* and cytochrome c (*Cyt-c*) genes are listed in Table 1.

Total RNA was extracted from cells with TRIzol and reverse-transcribed with a cDNA synthesis kit (TransGen Biotech Co., Ltd., Beijing, China). Quantitative real-time PCR (RT-qPCR) with SYBR green as a fluorescent probe was used to determine the mRNA levels. A 480 real-time PCR system (Roche LightCycler, Basel, Switzerland) was used for analysis. The 2^−ΔΔCT^ method was used to calculate the mRNA levels of the target genes [19]. The mRNA levels of the analyzed genes in the samples were normalized to the expression level of glyceraldehyde-3-phosphate dehydrogenase (GAPDH), which served as the reference gene. The control group consisted of untreated cells.

### 2.11. Statistical Analysis

All the data were expressed as the means ± standard deviations of the means. The Student’s *t*-test for single comparisons and one-way ANOVA for multiple comparisons were used to determine the statistical significance of the differences between the means. The significance of the results was determined at the *p* < 0.05, *p* < 0.01, *p* < 0.001, and *p* < 0.0001 levels. GraphPad Prism 8 (GraphPad Prism, San Diego, CA, USA) was used for all the statistical calculations and plots.

## 3. Results

### 3.1. Total Lipid and Protein Contents of DaMe

The total lipid and protein contents of DaMe were found to be 6.1% and 3.4%, respectively.

### 3.2. Fatty Acid Composition and Element Content of DaMe

Among the 12 fatty acid substances screened, DaMe had the highest content of palmitic acid (27.8%) and the lowest content of butyric acid (0.8%) according to the analysis results obtained with a gas chromatography–flame ionization detector (GC–FID) device and presented in the Supplementary Materials as Table S1.

**Figure 1:**
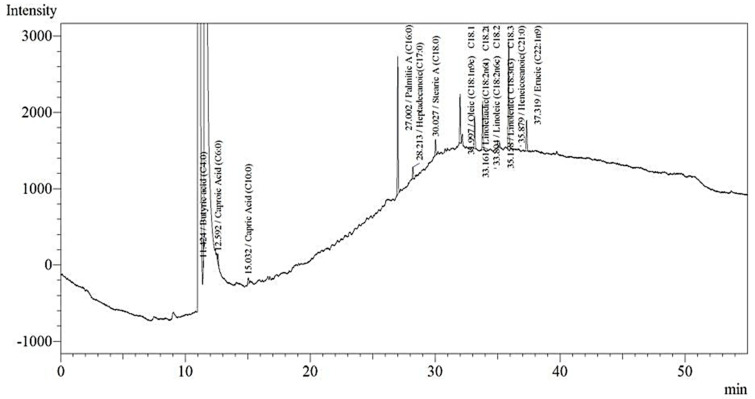
GC-FID chromatogram of DaMe.

The peaks obtained for each fatty acid in the GC-FID chromatogram are shown in Figure 1.

According to the ICP-MS analysis results, the most abundant element in DaMe is sodium (Na = 1375.9 ppm), and the least abundant element is selenium (Se = 0.33 ppm) (Supplementary Materials, Table S2).

### 3.3. Effect of DaMe on ROS Production in SH-SY5Y Cells Induced by 6-OHDA

**Figure 2:**
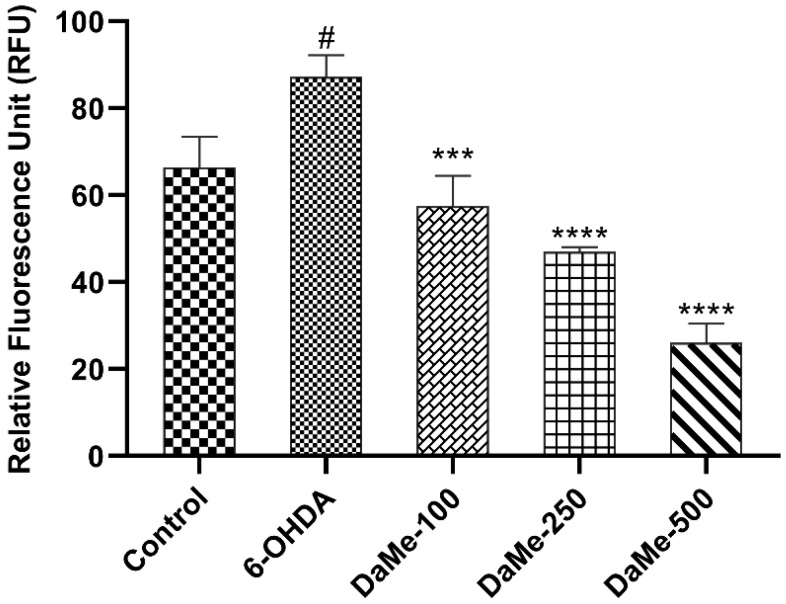
Effect of DaMe pretreatment on ROS production in 6-OHDA-induced SH-SY5Y human neuroblastoma cells. The RFU results demonstrated the dose-dependent reduction in the ROS levels following the DaMe pretreatment, indicating its protective effect against oxidative stress induced by 6-OHDA. ^#^ *p* < 0.05, *** *p* < 0.001, **** *p* < 0.0001.

Following a 24 h treatment with 150 µM of 6-OHDA, the production of ROS by oxidative stress in SH-SY5Y human neuroblastoma cells was assessed. The fluorescence results showed that the cells treated with 6-OHDA (87.2 RFU) produced significantly more ROS than the control group (66.3 RFU) (*p* < 0.05). This increase was mitigated in a dose-dependent manner by pretreatment with DaMe at concentrations of 100, 250, and 500 µg/mL, which decreased the increase to 57.4, 46.9, and 26 RFU (*p* < 0.001, *p* < 0.0001, and *p* < 0.0001), respectively (Figure 2).

### 3.4. Effect of DaMe on the Oxidative Stress in SH-SY5Y Cells Induced by 6-OHDA

**Figure 3:**
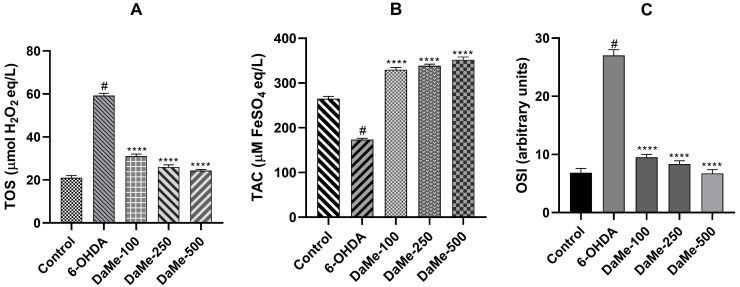
The effects of DaMe on total oxidant status (TOS) (**A**), total antioxidant capacity (TAC) (**B**), and oxidative stress index (OSI) (**C**) in 6-OHDA-treated SH-SY5Y cells. DaMe pretreatment significantly reduced TOS and OSI levels while increasing TAC levels, illustrating its dual role in mitigating oxidative stress and enhancing antioxidant defense mechanisms. ^#^ *p* < 0.05 and **** *p* < 0.0001.

To investigate the possible involvement of oxidative stress modulation in the neuroprotective effects of DaMe, the levels of the TOS and TAC were determined spectrophotometrically. Treatment with 6-OHDA led to higher TOS levels (*p* < 0.05) and lower TAC levels (*p* < 0.05) in comparison to those of the control. When the DaMe was pretreated at concentrations of 100, 250, and 500 µg/mL to SH-SY5Y cells prior to 6-OHDA toxicity, the TOS levels significantly decreased (*p* < 0.0001) (Figure 3A), whereas the TAC levels increased (*p* < 0.0001), suggesting a dose-dependent effect on oxidative stress (Figure 3B). Accordingly, at the DaMe concentrations, the OSI values in the SH-SY5Y cells were significantly reduced (*p* < 0.0001) (Figure 3C).

### 3.5. Effect of DaMe on the Mitochondrial Membrane Potential of SH-SY5Y Cells Induced with 6-OHDA

**Figure 4:**
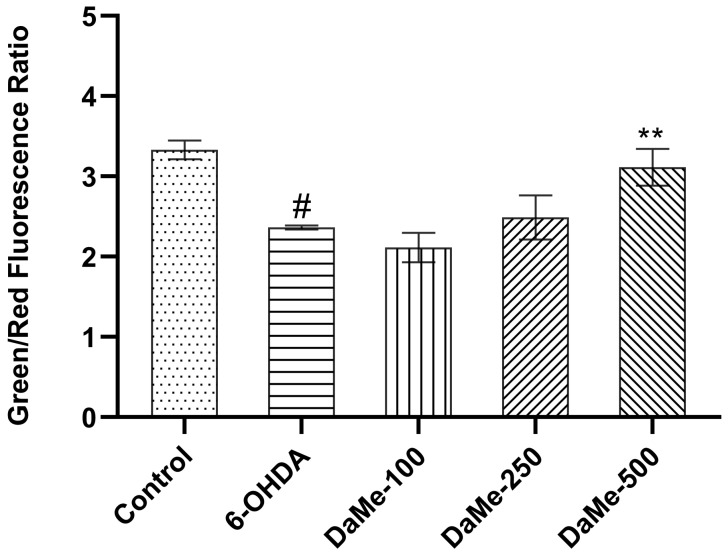
Effect of DaMe pretreatment on mitochondrial membrane potential (ΔΨm) in 6-OHDA-induced SH-SY5Y human neuroblastoma cells. DaMe at the 500 µg/mL concentration showed a protective effect on the mitochondrial integrity against damage caused by 6-OHDA by the restoration of the ΔΨm. ^#^ *p* < 0.05 and ** *p* < 0.01.

The oxidative stress-related ΔΨm of SH-SY5Y human neuroblastoma cells was measured after the cells were treated with 150 µM of 6-OHDA for 24 h. The fluorescence data showed that cells treated with 6-OHDA for 24 h had a considerably lower red/green fluorescence ratio (ΔΨm) of 2.3 (*p* < 0.05) than that of the control group, which had a ratio of 3.3. After pretreatment with DaMe at concentrations of 100, 250, and 500 µg/mL, the ΔΨm was significantly increased in the DaMe concentration of 500 µg/mL (*p* < 0.01) (Figure 4).

### 3.6. Effect of DaMe on Oxidative DNA Damage in SH-SY5Y Cells Induced with 6-OHDA

**Figure 5:**
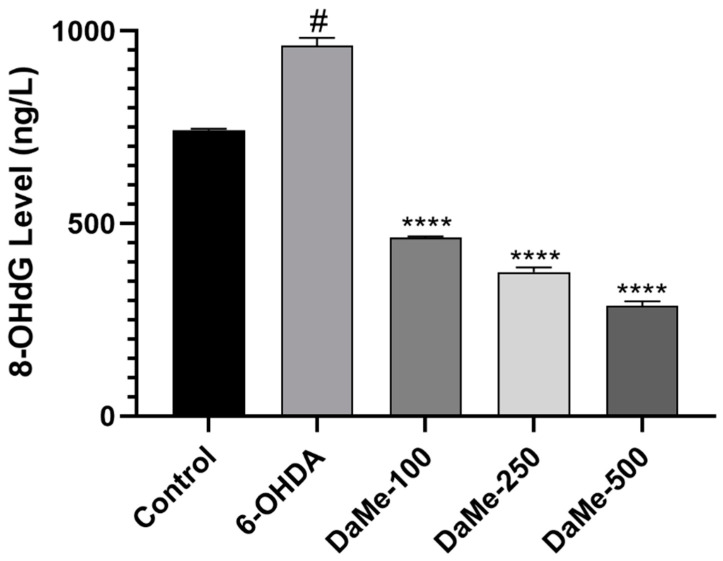
Effect of DaMe pretreatment on 8-OHdG levels in 6-OHDA-induced SH-SY5Y human neuroblastoma cells. DaMe pretreatment significantly reduced 8-OHdG levels, suggesting its potential to prevent oxidative DNA damage caused by 6-OHDA. ^#^ *p* < 0.05 and **** *p* < 0.0001.

Following the 24 h pretreatment with DaMe at concentrations of 100, 250, and 500 µg/mL, the 8-OHdG levels were measured in the 6-OHDA-induced SH-SY5Y cells. The DaMe prevented oxidative DNA damage by reducing the 8-OHdG levels in the 6-OHDA-induced SH-SY5Y cells. The level of 8-OHdG in the 6-OHDA-induced group (962.7 ng/L) was significantly higher (*p* < 0.05) than that in the control group (742.8 ng/L), while it decreased in a dose-dependent manner in the DaMe-pretreated groups (464.048, 374.65, and 286 ng/L for 100, 250, and 500 μg/mL DaMe concentrations, respectively; *p* < 0.0001) (Figure 5).

### 3.7. Effect of the DaMe on Expressions of Parkinson’s Disease and Apoptosis-Related Genes in 6-OHDA-Induced SH-SY5Y Cells

**Figure 6:**
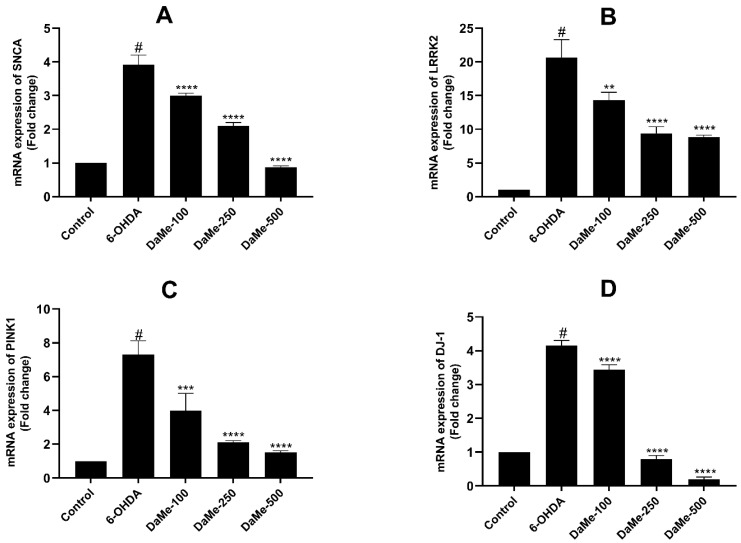
Effect of DaMe pretreatment on *SNCA* (**A**) *LRKK2*, (**B**) *PINK1*, (**C**) and *DJ-1* (**D**) gene expressions in 6-OHDA-induced SH-SY5Y human neuroblastoma cells. DaMe pretreatment significantly downregulated PD-related gene expressions, indicating that it modulates genes associated with oxidative stress and neuronal damage. ^#^ *p* < 0.05, ** *p* < 0.01, *** *p* < 0.001, and **** *p* < 0.0001.

The effect of DaMe on SH-SY5Y cells treated with 150 µM of 6-OHDA was investigated at the mRNA level. The mRNA levels of the Parkinson’s-related *SNCA*, *LRRK2*, *PINK1*, *DJ-1*, and *PARK2* genes and the apoptosis-related *p53* and *Cyt-c* genes were determined by RT-qPCR analysis. After the 6-OHDA treatment, there were significant increases of 3.9-, 20.7-, 7.3-, and 4.1-fold in the mRNA levels of the *SNCA*, *LRKK2*, *PINK1,* and *DJ-1* genes, respectively (*p* < 0.05) (Figure 6). A 24 h pretreatment with DaMe at concentrations of 100, 250, and 500 µg/mL of 6-OHDA-induced SH-SY5Y cells decreased the mRNA levels of the *SNCA* gene (3-, 2.1-, and 0.9-fold changes, respectively; *p* < 0.0001), *LRRK2* gene (14.3-, 9.3-, and 8.8-fold changes, respectively; *p* < 0.01, *p* < 0.0001, and *p* < 0.0001), *PINK1* gene (3.9-, 2.1-, and 1.5-fold changes, respectively; *p* < 0.001, *p* < 0.0001, and *p* < 0.0001), and *DJ-1* gene (3.4-, 0.8-, and 0.2-fold changes, respectively; *p* < 0.0001).

**Figure 7:**
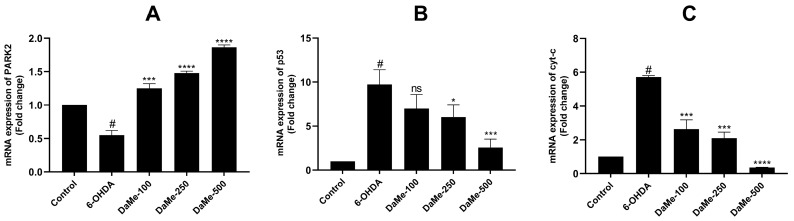
Effect of DaMe pretreatment on *PARK2* (**A**), *p53* (**B**), and *Cyt-c* (**C**) gene expressions in 6-OHDA-induced SH-SY5Y human neuroblastoma cells. DaMe pretreatment significantly upregulated PARK2 gene expression and downregulated apoptotic gene expression, indicating its neuroprotective and antiapoptotic effects. ^#^ *p* < 0.05, * *p* < 0.05, *** *p* < 0.001, and **** *p* < 0.0001, ns means not significant.

Following the 6-OHDA treatment, the *PARK2* gene expression level decreased 0.5-fold (*p* < 0.05), but the expression levels of the *p53* and *Cyt-c* genes increased approximately 9.7- and 5.7-fold, respectively (Figure 7). A 24 h pretreatment with DaMe at concentrations of 100, 250, and 500 µg/mL of 6-OHDA-induced SH-SY5Y cells upregulated the mRNA level of the *PARK2* gene (1.25-, 1.48-, and 1.84-fold changes; *p* < 0.001, *p* < 0.0001, and *p* < 0.0001, respectively) and downregulated the *p53* gene (6.9-, 6-, and 2.5-fold changes; *p* > 0.05, *p* < 0.05, and *p* < 0.001, respectively) (Figure 7). The decrease in the *p53* gene expression at the 100 µg/mL DaMe concentration was not statistically significant. All concentrations of DaMe significantly downregulated the expression level of the *Cyt-c* gene compared with that in the 6-OHDA group (2.6-, 2.1-, and 0.3-fold changes; *p* < 0.001, *p* < 0.001, and *p* < 0.0001, respectively) (Figure 7).

## 4. Discussion

PD is a progressive neurodegenerative disorder characterized by the loss of dopaminergic neurons and the presence of motor symptoms such as tremors, rigidity, and bradykinesia. While the pathogenesis of PD is not yet fully understood, both genetic and environmental factors play significant roles in the disease development [20]. In this context, mitochondrial dysfunction and oxidative stress are key contributors to the progression of PD [21]. Mitochondrial functions are crucial for cellular energy production and metabolism. PD is associated with mitochondrial dysfunction, which disrupts energy production and increases oxidative stress, forming free radicals that damage cellular components such as DNA, proteins, and lipids [22]. This process particularly affects sensitive cell types such as dopaminergic neurons, contributing to the disease’s progression. The role of oxidative stress in the development of PD has led to the exploration of new therapeutic strategies targeting oxidative damage. By addressing the mechanisms of oxidative stress, potential treatments may slow the disease.

This study investigated the potential of DaMe in alleviating the biological effects of PD. DaMe is believed to reduce oxidative stress and protect mitochondrial functioning [14]. Its biological properties make it an attractive candidate for investigating its potential therapeutic effects against PD. This study assessed the protective effects of DaMe in an in vitro model of PD produced by 6-OHDA in SH-SY5Y human neuroblastoma cells. 6-OHDA, which is widely used to create a model that mimics the pathology of PD, is a catecholaminergic neurotoxin that stimulates ROS production in neuronal cells, leading to oxidative stress and cell death [6,23].

Quercetin, one of the best-known flavonoids, is present in large amounts in vegetables and fruits. It has many health benefits, including anti-obesity, anti-carcinogenic, anti-inflammatory, and antibacterial effects [24]. According to our previous study’s results [14], the amount of quercetin, which was the only detectable amount among the seven phenolic compounds studied, was very low compared to previous studies [25,26]. Therefore, other flavonoids contributing to the total phenolic content should also be explored in DaMe.

Although there are studies on the relationship between microalga molecules such as pigments, lipids, polysaccharides, and proteins and diseases, as far as we know, there are no studies in the literature on the evaluation of the active pharmaceutical ingredients directly contained in the extracts. Our previous study’s results [14] showed that the most abundant drug constituent of DaMe was diclofenac, a widely preferred non-steroidal anti-inflammatory drug (NSAID). Although some studies suggest that diclofenac causes apoptosis in SH-SY5Y cells by altering mitochondrial functioning [27], a recent study has shown that its combination with other agents enhances neuroprotective effects [28]. Because pretreatment with DaMe suppressed rather than induced apoptosis, we hypothesize that the combination of diclofenac and other unidentified components in our extract may have a stronger effect than the effect alone, taking into account the aforementioned studies and our findings. This phenomenon may be explained by the fact that a substance’s mode of action is more significant than its quantity.

The second most common active ingredient in DaMe was the psychostimulant medication methylenedioxymethamphetamine (MDMA) [14]. MDMA is a synthetic drug that raises serotonin, norepinephrine, and dopamine levels in the brain. According to certain earlier research, UWA-101, an MDMA analog, lessens L-DOPA’s negative effects without obstructing its positive ones [29,30]. However, in animal models, this medication is neurotoxic in a number of brain regions. There is not enough evidence to support the growing consensus that MDMA can be neurotoxic to humans [31]. Given that our study demonstrated the neuroprotective potential of MDMA, we suspect that other molecules in DaMe may have suppressed it. DaMe needs more research in various PD models.

Our findings align with those of previous studies demonstrating the neuroprotective potential of natural products against 6-OHDA-induced neurotoxicity. For example, studies on other microalgal species, such as *Chlorella vulgaris* and *Spirulina platensis*, have shown their ability to reduce oxidative stress and improve mitochondrial functioning in similar in vitro models [32,33,34,35]. To the best of our knowledge, this is the first study to investigate the neuroprotective effect of the extract of *Desmodesmus arthrodesmiformis* in SH-SY5Y cells exposed to 6-OHDA. This study focused on the effects of the methanol extract of *Desmodesmus arthrodesmiformis* EM13 (DaMe) on oxidative stress parameters such as the TAC, TOS, OSI, ROS production, 8-OHdG levels, and mitochondrial membrane potential in SH-SY5Y cells induced with 6-OHDA. In addition, changes in the expression levels of the Parkinson’s-related *SNCA*, *LRRK2*, *PINK1*, *DJ-1*, and *PARK2* genes and the apoptosis-related *p53* and *Cyt-c* genes were investigated.

Elevated ROS are among the possible cellular causes of PD and other neurodegenerative diseases. The mitochondrial membrane potential is a commonly used parameter for determining mitochondrial health. Studies indicate that natural products, including methanol extracts, can reduce ROS levels and restore mitochondrial functioning, which are critical for preventing neuronal apoptosis [36,37,38]. In our study, in agreement with the results of previous studies [39], the treatment of human SH-SY5Y neuroblastoma cells with 6-OHDA increased the ROS production in an in vitro model of PD. However, our study revealed that DaMe reduced the intracellular ROS levels, which were impaired by 6-OHDA damage. Our findings also revealed that DaMe decreased the TOS and OSI levels and increased the TAC levels by approximately two-fold in an in vitro model of PD. Given these results, DaMe effectively reactivated the antioxidative mechanisms that the 6-OHDA treatment had suppressed, indicating that it may have neuroprotective properties. Furthermore, our results revealed that the mitochondrial membrane potential (ΔΨm) decreased in an in vitro model of PD, and that pretreatment with DaMe before treatment with 6-OHDA increased it. These results might show clear signals of DaMe’s mitochondrial protection. In contrast to previous studies reporting increased mitochondrial membrane potential solely through antioxidant mechanisms [40], our results indicate that DaMe not only restores the ΔΨm but also modulates apoptotic and mitophagic pathways, highlighting its multifaceted protective effects. This broader mechanism of action suggests that DaMe may be more advantageous in therapeutic applications than single-pathway antioxidants.

The present study found that in an in vitro model of PD, the pretreatment of cells with DaMe before exposure to 6-OHDA greatly reduced the 8-OHdG levels, a marker of oxidative DNA damage. Similar to the effects of DaMe in our study, extracts from *Hypericum perforatum* and *Crataegus* spp. have also been shown to reduce 8-OHdG levels and ROS production [41,42].

There is strong evidence that mutations in the *SNCA*, *LRRK2*, *PINK1*, and *DJ-1* genes cause familial PD, even if the precise nature of the condition is still unknown [43]. These genes encode proteins that are closely associated with the mitophagy process and, thus, with the regulation of neuronal survival. Following an oxidative stimulus, α-synuclein (α-syn) proliferates in neurons. The misfolding and aggregation of α-syn can even lead to the formation of Lewy bodies and the degeneration of the brain that are characteristic of PD. As of yet, the exact mechanisms underlying the toxicity of α-syn remain unclear. Intermittent PD that results in dementia is exacerbated by oxidized α-syn [44,45]. In our study, the α-syn (*SNCA*) gene, which produces the protein, was downregulated after pretreatment with DaMe in an in vitro model of PD. Moreover, the overexpression of α-syn leads to an increase in ROS production at the cellular level and initiates a vicious cycle in which α-syn induces ROS and vice versa [46]. Recently, FAM171A2 has been recognized as a potential receptor involved in the neuronal uptake of α-syn fibrils, making it a promising therapeutic target for PD [47]. Our study focuses on mitochondrial oxidative stress mechanisms that are not directly related to α-synuclein internalization or FAM171A2 functioning. However, DaMe may show promise in treating PD, potentially by affecting the FAM171A2-α-syn pathway.

Gain-of-function mutations in the *LRRK2* gene have been shown to increase the susceptibility of neurons to oxidative stress. By altering the levels of its protein at its site of action, LRRK2 is closely linked to the pathological appendages of several neurodegenerative diseases [48]. However, the extent to which LRRK2 contributes to PD pathology is not yet known. In our study, the oxidative stress induced by 6-OHDA led to an expected strong increase in *LRRK2*; this effect was reversed by pretreatment with DaMe. Since *LRRK2* has been identified as a pathology-associated protein in PD, the reduction in the *LRRK2* gene expression by DaMe in an in vitro model of PD may indicate that it may be a neuroprotective agent.

Parkin and its regulator *PINK1* are known to play a role in mitochondrial survival and protection against ROS and to homodimerize together with *DJ-1*, representing another antioxidant neuronal defense [3]. Thus, *PINK1* maintains mitochondrial homeostasis and provides neuroprotection. In our study, pretreatment with DaMe led to the downregulation of the *PINK1* gene expression in an in vitro model of PD in which ROS generation and the relative upregulation of the *PINK1* gene balanced the oxidative reactions and mitochondrial instability within the cell. In contrast, *DJ-1* is thought to have various functions, including mitochondrial regulation, transcriptional regulation, chaperone activity, protease activity, and the antioxidant stress response. By oxidizing its cysteine residue at position 106, the gene product protein park7 shows activity related to its oxidative status. Furthermore, excessive *DJ-1* oxidation masks *DJ-1* activity, which is more pronounced in people with sporadic PD. *DJ-1* quenches ROS via the self-oxidation of cysteine residues [49,50]. In our study, *DJ-1* was overexpressed in cells exposed to 6-OHDA but returned to normal when pretreated with DaMe.

Additionally, several studies have shown that flavonoid-rich extracts of plant sources, such as mandarin juice and glycyrrhizic acid, have gene regulatory effects comparable to the modulation of PD-related genes, such as *SNCA*, *PINK1*, and *DJ-1*, by DaMe [51,52,53]. These results may suggest that the mechanisms by which bioactive compounds from various natural sources—including microalgae—provide neuroprotection are similar.

Cytochrome c release from mitochondria is an important mechanism for activating caspase-3 and the induction of cell death in response to ‘intrinsic’ pro-apoptotic stimuli, including oxidative stress. 6-OHDA induces cytochrome *c* release from mitochondria in SH-SY5Y cells [54]. The tumor suppressor p53 is sensitive to stress such as DNA damage and hypoxia, and it is activated by its phosphorylation and acetylation, triggering cell cycle arrest and apoptosis [55]. 6-OHDA induces cytochrome *c* release from mitochondria in SH-SY5Y cells. In our study, DaMe downregulated the rise in the *p53* and *Cyt-c* gene expressions induced by the stressor 6-OHDA. The reductions in these apoptotic gene expressions may reveal that the antiapoptotic effect and oxidative stress status reduction result from the preventive effects of microalga extracts.

While the observed effects of DaMe are promising, its exact bioactive compounds and their pharmacodynamics remain unexplored. This limitation mirrors challenges faced in other natural product studies where the complexity of the extracts makes precise mechanism elucidation difficult. Future studies focusing on isolating these compounds and comparing their effects with those of purified bioactive agents will be crucial. The other limitation is that it would be very difficult to deliver DaMe to mitochondria in vivo. However, several strategies have been explored to enhance the mitochondrial localization of therapeutic compounds, including mitochondria-targeting peptides, lipophilic cations (such as TPP+ derivatives), and nanoparticle-based delivery systems.

## 5. Conclusions

DaMe effectively prevented the oxidative stress caused by 6-OHDA in a cellular model of PD. It protected cells from excessive ROS production by targeting mitochondrial damage pathways through mechanisms involving ROS reduction, oxidative stress modulation, and mitochondrial membrane potential stabilization. Furthermore, DaMe increased cell survival by inhibiting apoptotic processes. In addition, although our study highlights the potential of DaMe for neuroprotection, it does not identify the specific bioactive components responsible for these effects. Future research should focus on isolating and characterizing these bioactive compounds. Studies exploring mitochondrial dynamics, such as mitophagy and bioenergetics, are recommended to better elucidate the underlying protective mechanisms of DaMe.

Moreover, developing DaMe-based formulations, such as nanoencapsulation, could increase their bioavailability and therapeutic potential in clinical settings. Investigating the synergistic effects of DaMe in combination with the existing pharmacological treatments for PD may also provide new insights into integrated therapeutic approaches. From a molecular perspective, DaMe partially restored the expression of factors related to mitochondrial functionality, the balance of which is disrupted by 6-OHDA and is critical for the clinical outcomes of PD. DaMe has strong neuroprotective effects in vitro and may indicate a potential therapeutic effect in PD; however, these findings need to be validated by in vivo and clinical studies.

## Figures and Tables

**Table 1:** Primers used in the RT-qPCR analysis.

Primer	Forward (F) and Reverse (R) Sequences (5′ → 3′)	NCBI Accession Number
*SNCA*	F: TGACAAATGTTGGAGGAGCA	NM_000345.4
	R: TGTCAGGATCCACAGGCATA	
*LRRK2*	F: TCAGCTTGTTGTTGGACAGC	NM_198578.4
	R: ACTGCGTGAGGAAGCTCATT	
*PINK1*	F: ACGTTCAGTTACGGGAGTGG	NM_032409.3
	R: GGCTAGTCAGGAGGGAAACC	
*DJ-1*	F: GGGTGCAGGCTTGTAAACAT	NM_007262.5
	R: GGACAAATGACCACATCACG	
*PARK2*	F: CTGACACCAGCATCTTCCAG	NM_004562.3
	R: CCAGTCATTCCTCAGCTCCT	
*P53*	F: TGACACGCTTCCCTGGATTG	NM_001126114.3
	R: GCTCGACGCTAGGATCTGAC	
*Cyt-C*	F: ACAAAGGCATCATCTGGGGAG	NM_018947.6
	R: AAGGCAGTGGCCAATTATTACTC	
*GAPDH*	F: GACAGTCAGCCGCATCTTCT	NM_002046.7
	R: GCGCCCAATACGACCAAATC	

## Data Availability

The data presented in this study are available upon request from the corresponding author.

## Supplementary Materials

The following supporting information can be downloaded at: https://www.mdpi.com/article/10.3390/cimb47030174/s1, Table S1: Fatty acid composition of DaMe determined by GC-FID analysis; Table S2: Elemental content of DaMe.
