# Supplementary material for: Desmodesmus Extract as a Mitochondrion-Targeted Neuroprotective Agent in Parkinson’s Disease: An In Vitro Study
Source: Curr Issues Mol Biol. 2025 Mar 6;47(3):174. doi: 10.3390/cimb47030174 (PMC11941442; doi:10.3390/cimb47030174)
Supplement: Supplementary file 1 [file cimb-47-00174-s001.zip › cimb-3504473-supplementary.pdf]

Table S1. Fatty acid composition of DaMe determined by GC-FID analysis

Peak number	Fatty acid (formula)	Retention time	Area	Area (%)
1	Butyric acid (C4:0)	11.424	216	0.8381
2	Caproic acid (C6:0)	12.592	334	1.2977
3	Capric acid (C10:0)	15.032	340	1.3229
4	Palmitic acid (C16:0)	27.002	7160	27.8312
5	Heptadecanoic acid (C17:0)	28.213	634	2.4645
6	Stearic acid (C18:0)	30.027	868	3.3754
7	Oleic acid (C18:1n9)	31.997	2745	10.6688
8	Linolelaidic acid (C18:2 trans-9,12)	33.161	1938	7.5351
9	Linoleic acid (C18:2n6)	33.804	2848	11.0699
10	Linolenic acid (C18:3n3)	35.158	290	1.1270
11	Heneicosanoic acid (C21:0)	35.879	6426	24.9784
12	Erucic acid (C22:1n9)	37.319	1927	7.4910
**Total**			25726	100.00

Table S2. Elemental content of DaMe

Element	Quantity (ppm)
Na	1375.9697
Mg	596.622451
K	862.336239
Ca	26.1925179
Mn	29.9784802
Fe	104.145005
Co	0.96869428
Zn	9.6535131
Se	0.33455171
Mo	6.2110678
